# Pulmonary and Respiratory Muscle Function in Response to Marathon and Ultra-Marathon Running: A Review

**DOI:** 10.1007/s40279-019-01105-w

**Published:** 2019-04-27

**Authors:** Nicholas B. Tiller

**Affiliations:** Academy of Sport and Physical Activity, Sheffield Hallam University, Collegiate Crescent, Sheffield, S10 2BP UK

## Abstract

The physiological demands of marathon and ultra-marathon running are substantial, affecting multiple body systems. There have been several reviews on the physiological contraindications of participation; nevertheless, the respiratory implications have received relatively little attention. This paper provides an up-to-date review of the literature pertaining to acute pulmonary and respiratory muscle responses to marathon and ultra-marathon running. Pulmonary function was most commonly assessed using spirometry, with infrequent use of techniques including single-breath rebreathe and whole-body plethysmography. All studies observed statistically significant post-race reductions in one-or-more metrics of pulmonary function, with or without evidence of airway obstruction. Nevertheless, an independent analysis revealed that post-race values rarely fell below the lower-limit of normal and are unlikely, therefore, to be clinically significant. This highlights the virtue of healthy baseline parameters prior to competition and, although speculative, there may be more potent clinical manifestations in individuals with below-average baseline function, or those with pre-existing respiratory disorders (e.g., asthma). Respiratory muscle fatigue was most commonly assessed indirectly using maximal static mouth-pressure manoeuvres, and respiratory muscle endurance via maximum voluntary ventilation (MVV_12_). Objective nerve-stimulation data from one study, and others documenting the time-course of recovery, implicate peripheral neuromuscular factors as the mechanism underpinning such fatigue. Evidence of respiratory muscle fatigue was more prevalent following marathon compared to ultra-marathon, and might be a factor of work rate, and thus exercise ventilation, which is tempered during longer races. Potential implications of respiratory muscle fatigue on health and marathon/ultra-marathon performance have been discussed, and include a diminished postural stability that may increase the risk of injury when running on challenging terrain, and possible respiratory muscle fatigue-induced effects on locomotor limb blood flow. This review provides novel insights that might influence marathon/ultra-marathon preparation strategies, as well as inform medical best-practice of personnel supporting such events.

## Key Points

**Table:** 

Pulmonary function (via spirometry) has been widely assessed following marathon and ultra-marathon, as has respiratory muscle fatigue (indirectly, via maximal static mouth-pressure manoeuvres).
Both event types are sufficient to provoke post-race decreases in pulmonary function in the range of 10–15% (with or without evidence of airway obstruction), and respiratory muscle fatigue in the range of 15–25%.
Post-race decreases in pulmonary function rarely reach clinical significance (i.e., values tend to remain within the lower-limits of normal), but implications may be more severe for individuals with pre-existing respiratory disorders or below-average baseline function.

## Introduction

The marathon footrace (42.2 km) was once considered among the greatest feats of human endurance [1] and, as a result, marathon running has been used widely as a model to investigate the limits of physiological function. The last few decades have seen an increased popularity of the ultra-marathon; defined as any footrace that exceeds the traditional marathon [2] and that typically lasts for > 6 h [3]. Most ultra-marathons take place over distances ranging from 50 to 160 km in a single stage, and up to 3100 km in multi-stage events. Races are contested the world over, often in remote locations and in extremes of temperature and altitude. The physiological demands of marathon and ultra-marathon events are substantial, affecting multiple body systems, thus providing an excellent model with which to study the adaptive responses to extreme load and stress [2].

In healthy humans, the respiratory system is considered to have sufficient capacity to meet the endurance exercise demands on pulmonary ventilation and gas exchange. For example, exercise ventilation of untrained subjects demands < 10% of both maximal oxygen uptake (V̇O_2max_) and maximum cardiac output (*Q̇*) [4, 5], and changes in intrathoracic pressure resulting from contractions of the respiratory muscles approximate only 40–50% of their pressure-generating potential [6]. Moreover, exercise-induced decreases in arterial O_2_ saturation are due predominantly to factors independent of the respiratory system (i.e., metabolic acidosis and hyperthermia, causing a rightward shift of the oxyhaemoglobin dissociation curve [7, 8]). Despite this apparent respiratory reserve, there are several mechanisms by which long-distance running might compromise pulmonary and/or respiratory-muscle function in healthy subjects. First, exercise is associated with airway cooling and/or dehydration and increased contact of the airway with polluting substances; consequently, potential activation of local and systemic inflammatory responses may result in airway obstruction, and negatively influence lung function (for review, see [9]). Second, marathon running has been associated with a mild, transient, post-race pulmonary oedema [10]. Although the prevalence and potential implications of pulmonary oedema are still debated, the phenomenon would be expected to compress small airways and blood vessels, affecting ventilation-perfusion matching [11]. Third, respiratory muscle work is a critical determinant of the magnitude of inspiratory muscle fatigue [12, 13], and the cardiorespiratory demands of a typical marathon (up to 75% V̇O_2max_ [14]) are likely sufficient to compromise respiratory muscle contractile function via reductions in Ca^2+^ availability in the sarcolemma [15]. Accordingly, there is now a growing body of work suggesting that the respiratory demands of prolonged endurance exercise may compromise resting pulmonary function, as well as attenuate the functional work capacity of the respiratory muscles, both of which might impact on health and endurance performance.

Despite several reviews on the applied physiology of endurance running, only one article published in the 1980s has reviewed the pulmonary responses to participation [1], concluding that marathon running is associated with moderate-to-high levels of pulmonary ventilation (> 80 L∙min^−1^), sufficient to provoke a reduction in post-race lung capacity, and evidence of respiratory muscle fatigue. The only contemporary review of the respiratory consequences of marathon running [16] offered a discussion of limited scope, and omitted most of the literature pertaining to indices of pulmonary function. In the last several decades, a number of new studies have reported decreased pulmonary function in response to marathon and ultra-marathon. Most studies have elucidated the time-course of recovery—there are reports on data collected both under controlled laboratory conditions and in the field, in remote and/or extreme environments, and several studies have assessed respiratory responses using advanced techniques such as whole-body plethysmography and magnetic nerve stimulation. Thus, we now have a more robust, mechanistic understanding of the maladaptive respiratory responses to marathon and ultra-marathon, and an updated review is warranted.

To date, there have been several reviews on the pathophysiology of ultra-marathon running [17–23]; yet, discussions of the respiratory implications are notably absent. For example, Knechtle and Nikolaidis [22] arguably provided the most comprehensive summary of ultra-marathon contraindications including: energetic demands; fluid and hydration; musculoskeletal damage; organ damage; and haematological, endocrine, cardiovascular, bone, digestive, and immune responses. The article, however, neglected to address the acute and/or chronic respiratory responses to competition. In a separate longitudinal analysis of medical issues among ultra-marathon runners [23], the prevalence of several respiratory-related disorders was discussed (e.g., lung disease, exercise-induced asthma, asthma, chronic bronchitis, pulmonary embolism, and spontaneous pneumothorax), but with no data presented on acute and/or chronic pulmonary or respiratory muscle function. Data to this effect would be helpful in the design of marathon and ultra-marathon training and preparation strategies, as well as in informing medical best-practice of personnel supporting the events (i.e., medics, race directors, and volunteers). This paper reviews the studies pertaining to pulmonary and respiratory muscle responses to marathon and ultra-marathon running, and discusses the potential implications of these responses on general health and race performance.

## Methods

This review deals with the responses of the pulmonary system (i.e., airflow and gas exchange) and the prevalence of respiratory muscle fatigue in marathon and ultra-marathon running. Although the marathon race is contested over a predetermined distance (i.e., 42.2 km), ‘ultra-marathon’ is a broad term comprising races of various distances, e.g., 31 miles (50 km; Blackwater Trail, Florida, USA) to 100 miles (161 km; Ultra-Trail du Mont-Blanc, Alps) in a single-stage, and up to 3100 miles (4989 km; Self-Transcendence 3100 Mile, New York, USA) in multi-stage events. Given the relative paucity of data on the acute respiratory responses to ultra-marathon running, there was no restriction on the race type considered for inclusion, with the caveat that the article assessed responses specifically to ultra-marathon (i.e., footraces) and not ultra-endurance racing in general. Articles were searched via three online databases (Pubmed, MEDLINE, Google Scholar; 1924–2019), and the search-terms comprised various combinations of the following: extreme-endurance; fatigue; lung; marathon; pathophysiology; physiology; pulmonary; respiratory; ultra-endurance; and ultra-marathon. The reference lists of those articles selected for inclusion were manually searched for additional literature.

### Inclusion/Exclusion Process

The following inclusion criteria were applied to the original abstracts retrieved using the above search-terms: (1) the study made a novel contribution to the literature on marathon or ultra-marathon (the latter defined as a race distance greater than a marathon) rather than ultra-endurance exercise in general; (2) the study explored aspects of pulmonary function and/or respiratory muscle function and/or respiratory muscle fatigue within the context of marathon or ultra-marathon running; (3) the study made comparable pre- and post-race assessments of one-or-more aspects of pulmonary and/or respiratory muscle function (irrespective of the time-course of the post-race measures); and (4) the article was available in English.

## Results

A total of 17 articles were originally found, two of which were unavailable in English and were, therefore, excluded from the summary (Table 1) and additional analyses. Of the 15 remaining articles, nine made assessments before/after a marathon, and six before/after an ultra-marathon; all were single-stage races except for one study investigating the responses to multiple, consecutive days of marathon running. A summary of the main findings of all 15 studies is shown in Table 1. With respect to pulmonary function, spirometry was the most widely utilised method (*n* = 12), with several assessments of diffusing capacity (*n* = 4) and intrathoracic pressures (*n* = 2). Respiratory muscle fatigue was widely assessed using volitional mouth-pressure manoeuvres (*n* = 6), with only one study using objective nerve stimulation techniques. Most studies compared baselines measures to those made immediately after the cessation of exercise (*n* = 10), with the remaining studies making assessments at varying post-race time-points (range 25 min - 3 days). Three articles made additional follow-up assessments at 24 h post-race. Finally, on those studies reporting a post-race decrease in one-or-more aspects of spirometry, additional independent analyses were conducted to elucidate if the reductions could be deemed *clinically* meaningful (i.e., fell below the lower-limits of normal; see “Discussion”).

**Table 1:** A chronological summary of the literature pertaining to pulmonary and respiratory muscle function in response to marathon and ultra-marathon running

Author (s)	Date	Event	Participants	Pre/post-race measures	Post-race timing
Gordon et al. [25]	1924	Marathon (42.2 km)	*N* = 22	FVC↓	< 5 min
Maron et al. [28]	1979	Marathon (42.2 km)	*N* = 13 (11 male)	FVC↓ FEV_1_↔ FEV_1_/FVC↑ FEF_200–1200_↓ RV↑ DL,_CO_↔	< 5 min; + 24 h
Mahler & Loke [29]	1981	Ultra-marathon (100 km)	*N* = 15	FVC↓ FEV_1_↓ FEV_1_/FVC↔ PEF↓ MEF_50_↓	< 15 min
Loke et al. [34]	1982	Marathon (42.2 km)	*N* = 4	FVC↔ FEV_1_↔ PEF↔ IC↔ MIP↓ MEP↓ P_di_,_IC_↓ MVV_12_↓	Data unavailable
Miles et al. [35]	1983	Marathon (42.2 km)	*N* = 8	FVC↔ FEV_1_↑ PEF↔ DL,_CO_↓ DM↓ CV↑	< 15 min
Warren et al. [37]	1989	Ultra-marathon (24 h)	*N* = 10	FVC↔ FEV_1_↔ PEF↔ IC↔ MIP↔ MEP↔ (MVV_12_↓ [after 24 h])	Data unavailable
Manier et al. [36]	1991	Marathon (42.2 km)	*N* = 11	DL,_CO_↓, DL_,NO_↓	~ 30 min
Chevrolet et al. [49]	1993	Marathon (42.2 km)	*N* = 21	MIP↓ MEP↔ (MVV_12_↔ [*N* = 15])	~ 2.5 h
Ker & Schultz [50]	1996	Ultra-marathon (87 km)	*N* = 10 (8 male)	MIP↔ MIP_TLim_↓	~ 3 d
Ross et al. [32]	2008	Marathon (42.2 km)	*N* = 9	FVC↔ FEV_1_↔ PIF↓ PEF↔ MIP↓ MEP↔	< 20 min; + 24 h
Zavorsky et al. [66]	2014	Marathon (42.2 km)	*N* = 36 (24 male)	FVC↓ FEV_1_↔ FEV_1_/FVC↑ PEF↔ FEF_25-75_↔ (DL,_CO_↔ DL,_NO_↔ DM,_CO_↓ [*N* = 24])	< 73 min
Wüthrich et al. [40]	2015	Ultra-marathon (110 km)	*N* = 22	FVC↔ FEV1↓ PIF↓ PEF↓ MIP↓ MEP↓ MVV_12_↓ (P_m,tw_↓ [*N* = 16])	< 90 min
Vernillo et al. [33]	2015	Ultra-marathon (330 km)	*N* = 29	FVC↓ FEV_1_↓ PIF↓ PEF↓ IC↔ TLC↓ RV↓ MVV_12_↓	< 5 min
Zavorsky et al. [39]	2019	Marathon (42.2 km)	*N* = 13	FVC↓ FEV_1_↓ PEF↓	~ 25 min
Tiller et al. [38]	2019	Marathon (42.2 km, × 10)	*N* = 9 (6 male)	FVC↔ FEV_1_↑ PEF↔ MIP↔ MEP↓	< 15 min

*FVC* forced vital capacity, *FEV*_*1*_ forced expiratory volume in 1 s, *FEF*_*200–1200*_ flow measured during the exhaled volume between 200–1200 mL of air, *RV* residual volume, *DL*,_*CO*_ diffusing capacity of the alveoli for carbon monoxide, *DL*,_*NO*_ diffusing capacity of the alveoli for nitric oxide, *PIF* peak inspiratory flow, *PEF* peak expiratory flow, *MEF*_*50*_ maximum expiratory flow at 50% of the FVC, *IC* inspiratory capacity, *MIP* maximum inspiratory pressure, *MEP* maximum expiratory pressure, *P*_*di,IC*_ peak inspiratory transdiaphragmatic pressure, *MVV*_*12*_ maximum voluntary ventilation, *DM* membrane diffusing capacity, *CV* closing volume, *TLim* time to the limit of tolerance, *P*_*m,tw*_ mouth twitch-pressure, *TLC* total lung capacity, *↑* increase, ↓ decrease, ↔ no change

## Discussion

### Pulmonary Function

Pulmonary function was most commonly assessed via spirometry. This physiological test assesses the competency with which an individual inspires or expires volumes of air as a function of time by requiring the subject to perform a series of forced vital capacity (FVC) manoeuvres into a mouthpiece; it is a valuable tool for screening general respiratory health [24]. An advantage of the technique is that spirometric assessments can be made both in the lab and in the field using portable equipment. The disadvantages, however, are that the FVC manoeuvre is volitional, and requires a degree of training and subject competency for reproducible data to be obtained.

The first study of pulmonary function in response to marathon running assessed lung capacity (FVC) in the first 22 finishers of the 1923 Boston Marathon, finding that the post-race values were significantly reduced by 0.8 L (17%) [25]. Several years later, pre- and post-race spirometric assessments revealed a fall in FVC of 0.7 and 0.9 L immediately following the Swiss marathons of 1927 and 1928, respectively [26, 27], thus corroborating the initial observations. Due to the absence of robust reference standards at the time of publication, it was difficult to contextualise these early data. Moreover, the mechanistic basis for the observations remained unexplored for several decades until 1979 when Maron et al. [28] tested spirometry and alveolar diffusing capacity for carbon monoxide (DL,_CO_) in 13 runners, before and immediately after the Wisconsin Mayfair Marathon; they also made follow-up assessments of the individual maximal expiratory flow-volume (MEFV) curves. They found that FVC was reduced by ~0.5 L (8.6%), attributable to an increase in residual volume (RV), but with no change in the forced expiratory volume in 1 s (FEV_1_) or the mean DL,_CO_. On assessing the post-race MEFV curves, the authors identified a diminished FEV in 1–2 seconds (FEV_1–2_), indicative of a reduction in small airway calibre at lower lung volumes, likely the result of an airway obstruction. This would be expected to permit premature closure of the airways and reduce FVC. Two years later, Mahler and Loke [29] observed a similar post-race fall in FVC (~0.6 L = 12.4%) with congruent reductions in FEV_1_ (9.5%) and peak expiratory flow (PEF; 13.7%) immediately following a 100-km footrace. The authors also observed a post-race drop in the maximal expiratory flow at 50% of FVC (MEF_50_), thereby supporting the notion that decreases in pulmonary function in this context might be attributable to small airway obstruction. The mechanisms underpinning this apparent airway obstruction are unclear, although they may be associated with a degree of airway inflammation (e.g., increased concentrations of polymorphonuclear neutrophils), as reported by Bonsignore et al. [30] following marathon running. Given that post-marathon nitric oxide (NO) concentrations were also elevated, the authors postulated that NO may have modulated the changes in exercise-associated airway inflammation [30]. Moreover, Araneda et al. [31] observed a greater relative increase in pulmonary pro-oxidative levels (hydrogen peroxide and nitrogen dioxide) following marathon running when compared to either half-marathon or 10-km running, thus suggesting that running time is positively associated with the magnitude of the acute post-exercise, pro-oxidative state. Nevertheless, to the author’s knowledge, the prevalence and magnitude of airway inflammation following ultra-marathon running (i.e., exercise of a longer duration and lower intensity) remain to be assessed.

Several of the studies on pulmonary function following distance running – Maron et al. (marathon) [28], Ross et al. (marathon) [32], and Mahler and Loke (ultra-marathon) [29] – made their assessments immediately after exercise, with follow-up measurements at 24 h post-race, by which time parameters had returned to baseline. Critically, both Mahler and Loke and Ross et al. made additional assessments at 2.5 h and 4 h post-race, respectively, by which point values had partially-recovered. Such a time-course of recovery (i.e., a transient decrease in function that recovers within a few hours), suggests that pulmonary function may have been influenced by a degree of respiratory muscle fatigue (see below).

More recently, Vernillo et al. used the absolute distance and unique terrain of extreme-endurance exercise as an excellent model to assess the adaptive potential of the human respiratory system. The researchers assessed spirometry before, during and immediately after a 330-km mountain ultra-marathon [33]. The main findings were congruent with the existing data, i.e., significant post-race decreases in FVC (9.5%), FEV_1_ (9.7%) and PEF (8.7%), but the ratios of FEV_1_/FVC, and FEV_1_/PEF were reasonably well-maintained pre-to-post race, thereby discounting lower- and upper-airway obstruction, respectively. Moreover, there was no evidence of airway obstruction on visual inspection of the MEFV curves. There was a significant post-race reduction in the maximum voluntary ventilation in 12 s (MVV_12_), which led the authors to attribute their findings, at least in part, to a fall in respiratory muscle endurance. Collectively, these studies suggest that both marathon and ultra-marathon running is sufficient to induce substantial, albeit highly varied, reductions in pulmonary function, with or without the presence of airway obstruction, and which are likely to be influenced by aspects of respiratory muscle fatigue.

There are several studies to the contrary, however, reporting limited evidence of a reduced pulmonary function following marathon or ultra-marathon running. In the early 1980s, Loke et al. [34] showed no change in pre-to-post-marathon vital capacity (VC), inspiratory capacity (IC) or FEV_1_. A year later, pre- and post-marathon spirometric assessments were supplemented by additional measures of pulmonary and membrane diffusing capacity (DL,_CO_ and DM, respectively) via the single-breath technique [35]. There was a post-race reduction in DL,_CO_ (observations that have been confirmed elsewhere [36]), as well as decreases in DM and closing volumes (CV) associated with a decrease in lung recoil, which the authors speculated may have been due to a modest degree of pulmonary oedema. Nevertheless, there was no associated influence on lung capacity or flow rate. Research by Zavorsky et al. observed a modest 2% decrease in post-marathon FVC, but no other changes in post-marathon indices of pulmonary function with the exception of a 12% decrease in alveolar membrane-diffusing capacity for carbon monoxide (DM,_CO_). When spirometry was performed by ten competitors at 3-h intervals throughout a 24-h ultra-marathon, there was a steady decline in lung function as the challenge progressed, but without any of the reductions reaching statistical significance [37]. Finally, in a ten-marathon stage-race we recently showed no consistent pre- to post-marathon decreases in FVC, FEV_1_, or PEF, thereby alluding to the robustness of the healthy respiratory system to respond to multiple consecutive days of endurance stimuli [38].

The published literature, therefore, shows equivocal findings with respect to pulmonary function following marathon and ultra-marathon running, but the reasons for such inconsistencies are unclear. There is considerable variation in the race distances and durations studied, including marathon (42.2 km, mean race time < 3 h 30 min), ultra-marathon ranging from 100 km (7 h 42 min) to 330 km (124 h 19 min) in a single stage, and multi-stage races of up to 400 km (46 h). Moreover, although the specifics of the race environment were not always reported, this likely varied in terms of both terrain and cumulative elevation which, in turn, would influence exercise intensity, and there was no consistency in the magnitude or prevalence of the observed decrease in pulmonary function. Furthermore, despite a range in athlete ability and experience among studies, there does not appear to be any correlation between the magnitude of the post-race fall in FVC and marathon finish time [28]. A recent study in which post-race spirometric values were reduced by 19–24% found no difference in the magnitude or prevalence of the change between males and females, or between subjects who completed a full- or half-marathon [39]. The available studies report airway obstruction intermittently, and although airway obstruction did appear to be slightly more common following ultra-marathon, the limited number of studies make this difficult to interpret. Interestingly, respiratory muscle fatigue was sometimes reported without a concomitant decline in lung function, thus reinforcing the notion that the two phenomena are independent, but a decrease in pulmonary function was always explained with at least some evidence of respiratory muscle fatigue, airway obstruction, or both. A further consideration is that there is variability with respect to the timing of post-race measures (range 5 min - 3 days). Exercise-induced decreases in pulmonary function are transient, and usually recover within a few hours of exercise cessation; as such, the literature is likely to be contaminated by non-significant findings from assessments made several hours or days post-race. Finally, respiratory responses were recorded following marathons contested in the summer [29, 37, 38, 40], in warm conditions [41], below freezing [41], or in a temperature-controlled laboratory [32], with no pattern in the post-race fall in pulmonary function. As a result, although decreased pulmonary function following marathon and ultra-marathon running is likely attributable to various combinations of airway obstruction, restriction, and respiratory muscle fatigue, further studies are needed to elucidate the specific external triggers for these mechanisms. Table 1 offers a summary of the pertinent studies and their main findings with respect to pulmonary function.

### Respiratory Muscle Fatigue

Respiratory muscle fatigue is a phenomenon whereby the inspiratory and/or expiratory muscles exhibit a reduced force-generating capacity relative to baseline measures [42]. Respiratory muscle fatigue has been assessed objectively using magnetic nerve stimulation following high-intensity, exhaustive cycling, running, and arm-cranking [12, 43, 44], and indirectly using maximal volitional mouth-pressure manoeuvres following swimming and rowing time-trials [45, 46]. Until the early 1980s, few studies had assessed the prevalence of respiratory muscle fatigue in response to marathon or ultra-marathon. There are now a number of reports of respiratory muscle fatigue under these conditions, the majority of which have used mouth-pressure manoeuvres as a surrogate for respiratory muscle force output, although several have also used MVV_12_ to estimate respiratory muscle endurance. Mouth-pressure manoeuvres are considered a global measure of respiratory muscle strength [47]. The technique is reproducible [48], non-invasive, can be reported alongside well-established normative data, and – critically for the study of marathon and ultra-marathon running – can be applied easily in the field using a handheld device. A common limitation of the technique is that manoeuvres are volitional, dependent on athlete motivation, and might be subject to a practice effect; these factors should be considered when interpreting the following data.

One of the first studies to assess respiratory muscle strength in response to competitive marathon reported a significant pre- to post-race fall in maximum inspiratory and expiratory mouth pressures of 16% and 28%, respectively [34]. The authors also reported a significant 20% fall in the peak transdiaphragmatic pressure elicited by IC manoeuvres, and a 10% reduction in respiratory muscle endurance assessed via MVV_12_. Although these data were obtained from a small sample (*n* = 4), the findings have since been corroborated in larger samples, in which similar reductions in maximum inspiratory pressure (~15%) were observed (*n* = 21 [49]; *n* = 9 [32]). It is worth noting that both studies measured, but found no evidence of, expiratory muscle fatigue.

By contrast, there were no significant changes in pre- to post-race respiratory muscle strength immediately following an 87-km ultra-marathon [50], or at any stage of a 24-h footrace during which assessments were made at 3-h intervals [37]; both studies observed post-race reductions in respiratory muscle endurance when assessed via inspiratory task-failure and MVV_12_, respectively. It should be noted that the post-race assessments in the former study by Ker et al. [50] were made 3 days after the event, by which time any transient effects of exercise on respiratory muscle contractile function would likely have abated; it is possible, therefore, that they underestimated the degree of respiratory muscle fatigue. In an effort to assess the presence of a cumulative reduction in resting respiratory muscle strength, we recently assessed maximum static mouth-pressures periodically throughout an ultra-marathon stage race comprising 10 marathons in 10 consecutive days [38]. Contrary to our hypothesis, there was no baseline drift in respiratory muscle function. Moreover, there was no post-marathon decrease in maximum inspiratory muscle strength, although there were persistent pre- to post-marathon decreases in maximum expiratory muscle strength. Collectively, these data suggest that inspiratory muscle fatigue is a common response to marathon running, whereas it may be that ultra-marathon running more readily provokes expiratory muscle fatigue.

Respiratory muscle work is a critical determinant of the magnitude of exercise-induced diaphragmatic fatigue [12, 13]. The running velocities typical of a single-stage marathon induce moderate-to-high levels of cardiorespiratory stress in the region of 60–75% V̇O_2_max, depending on trained status[14]. Moreover, participants in published studies have recorded mean race times of < 3 h 30 min [32, 34], thus representing the 91st percentile. As such, those subjects likely exhibited higher average ventilations than recreational runners. By contrast, ultra-marathons, particularly those of extreme distance, necessitate that runners temper their race intensity in order to prioritise endurance for the latter stages of the event. Throughout an ultra-endurance stage race [38], this notion of preservation manifested in lower ratings of post-marathon dyspnoea (~ 2.0, Borg CR10 scale) relative to those reported elsewhere during a single marathon competition (~ 12, Borg 6–20 scale [32]). As such, it may be that most long-distance ultra-marathons do not impose a sufficient ventilatory stimulus to provoke fatigue of the inspiratory muscles. By contrast, the major expiratory muscles (e.g., the rectus abdominis), adopt several non-ventilatory roles during exercise, including postural support [51] and static contractions to increase thoracic pressure to stiffen the spine [52]. Moreover, the specific demands of ultra-marathon running (e.g., uneven terrain, long durations, carrying weighted backpacks) likely increase postural loads on the abdominals, thereby rendering them more susceptible to fatigue. Although the diaphragm also has a postural role, this is only coordinated with its respiratory functions during transient, intermittent disturbances to trunk stability (e.g., brief arm movements) [53]. Moreover, when humoral factors mediate ventilation (e.g., during sustained exercise), postural drive to the phrenic motoneurons is withdrawn and respiratory input is prioritised [54]. A diminished postural drive to the diaphragm, coupled with a modest ventilatory demand, would be a likely explanation for the lack of inspiratory muscle fatigue thus far noted in response to ultra-marathon running.

When interpreting the data on respiratory muscle function following marathon and ultra-marathon running, the timescale of recovery is an important consideration because it offers an insight into the contributing mechanisms. Studies that have assessed maximum mouth pressures pre- and post-marathon or ultra-marathon have noted: (1) significant reductions immediately post-race [35, 38]; (2) significant reductions at 2.5 h post-race [49]; (3) partial recovery at 4 h post-race [32]; and (4) full recovery at 24 h post-race [32]. There are currently no published studies that report manifestations of respiratory muscle fatigue beyond 24 h post-race. Such a time-course of recovery is indicative of low-frequency, peripheral fatigue, the underlying mechanisms of which are thought to be reduced Ca^2+^ release from the sarcoplasmic reticulum, reduced Ca^2+^ sensitivity of the myofibrils, and/or damaged sarcomeres caused by overextension of the muscle fibres [55]. Given that values are lowest immediately after exercise, and appear to partially recover at 4 h, the fatigue observed was likely due to reduced calcium release and/or sensitivity.

To date, only one study has assessed respiratory muscle fatigue following ultra-marathon running using nerve stimulation techniques to artificially (and maximally) activate the respiratory muscles [40]. Direct induction of the phrenic nerves using this procedure is preferred to maximal static mouth-pressure manoeuvres because it results in an involuntary response from the respiratory muscles that is not influenced by participant motivation. Twenty-two experienced ultra-marathon runners completed a 110-km mountain race with ~ 6000 m of positive elevation. Assessments made within 90 min of the finish demonstrated a significant reduction in both maximum inspiratory and expiratory mouth pressures (16% and 21%, respectively), and a further 19% reduction in the inspiratory mouth twitch-pressure following cervical magnetic stimulation. Given that there were only minor changes in post-race voluntary activation of the inspiratory muscles (i.e., the ratio between superimposed twitch amplitude and the subsequent mouth twitch-pressure), the authors proposed that fatigue of the inspiratory muscles under these conditions was attributable to peripheral neuromuscular factors, as opposed to fatigue that was centrally mediated. The observation of a significant fall in the inspiratory mouth twitch-pressure challenges the existing ultra-marathon literature, which has largely failed to observe such a fatigue of the inspiratory muscles. One explanation is the nature of the race contested (Ultra-trail du Mont-Blanc, Alps) which has strict qualifying standards, high elevation gain, and necessitates runners of exceptional ability and experience. As such, it is likely that the race induced substantial and sustained pulmonary ventilation, above-and-beyond that observed in most other race types, thus, predicating the observed fatigue. Table 1 offers a summary of the pertinent studies and their main findings with respect to respiratory muscle function.

### Thoracic Load Carriage

Marathons contested in remote locations (e.g., in the mountains), and most ultra-marathons, require participants to run with backpacks that contain essential equipment including basic medical supplies, emergency clothing, and food. Several studies have assessed the influence of weighted backpacks on resting pulmonary function, observing significant reductions in both FVC and FEV_1_ [56–58]. In an effort to elucidate the mechanisms underpinning these findings, Dominelli et al. [59] used oesophageal balloon catheters to assess intrathoracic pressures and respiratory mechanics during treadmill walking with backpacks of different masses. Relative to un-weighted backpacks, and a no-pack control, there was a progressive reduction in both FVC and FEV_1_ with increasing pack weight (15, 25, and 35 kg), a finding attributed to ribcage restriction caused by changes in chest-wall compliance. A further observation was that heavier backpacks increased the work of breathing, although this was likely due to an increased minute ventilation second to elevated metabolic demands. Interestingly, there was no difference in FVC between the unweighted backpack and the no-pack control, suggesting that chest-straps alone do not influence lung capacity. Nevertheless, due to the presence of adequate ventilatory reserve, acute reductions in FVC are unlikely to hinder minute ventilation during exercise of a low ventilatory demand [59].

Notwithstanding the influence of weighted backpacks on pulmonary function, elevated thoracic loads may also influence the fatigability of respiratory muscles, although findings are inconsistent. Nadiv et al. [60] assessed the root mean square (RMS) amplitude and mean power frequency of the external intercostals and sternocleidomastoids during 1-h treadmill marching with, and without, a backpack weighing 15 kg. The load resulted in a significant increase in EMG, suggesting a degree of fatigue with a central component. Nevertheless, these observations do not necessarily translate to diminished function, given that submaximal walking with backpack loads ranging from 0 to 20 kg have no influence on parameters of respiratory muscle strength [61]. Sixty minutes of brisk walking with a heavy backpack (25 kg), however, significantly reduced inspiratory and expiratory muscle strength by 11% and 13%, respectively. Values were further reduced to 16 and 19%, respectively, following a subsequent 2.4-km time-trial [62]. Collectively, these data suggest that heavier thoracic loads (i.e., > 20 kg) are necessary to influence respiratory muscle function during prolonged exercise, although studies using nerve stimulation techniques are needed to objectively corroborate these findings. It is worthy of consideration that, while certain military training regimens and ultra-endurance expeditions might require marching and running with backpacks weighing > 20 kg, the majority of marathons and ultra-marathons do not; as such, the influence of weighted backpacks on the magnitude and prevalence of respiratory muscle fatigue during these race types is likely minimal.

### Implications for Health and Performance

There are several means by which reduced pulmonary function and/or respiratory muscle fatigue might impact on health or endurance running performance. First, the respiratory muscles have a critical role in maintaining torso stabilisation during exercise. The diaphragm, for example, contracts isometrically during repetitive postural tasks [63], and the major expiratory muscles contract to provide postural support [51] by increasing intra-abdominal pressure [52], thereby stiffening and stabilising the lumbar spine. This likely helps to protect spinal structures during periods of postural disturbance. As a consequence, exercise that induces expiratory muscle fatigue might place the runner at a greater risk of injury, and render them less able to sustain the rigours of competition. Moreover, when considering the substantial exercise-induced reductions in calf and quadriceps strength following ultra-marathon [64], a simultaneous respiratory and locomotor muscle fatigue may further increase the risk of fall and/or injury when traversing challenging terrain (e.g., a rocky or mountainous trail).

Second, the influence of lung function on marathon performance was recently assessed in a sample of 110 marathon runners [65]. Baseline spirometry was performed ~3 days prior to a competitive marathon, after which runners were subcategorised by finish time. There were significant negative correlations between indices of pulmonary function and marathon finish time; i.e., faster marathon runners exhibited better metrics of lung function (FVC; *r* = − 0.41, *p* < 0.001: FEV_1_; *r* = − 0.40, *p* < 0.001: PEF; *r* = − 0.50, *p* = 0.005). These data should be interpreted with care, inasmuch as there may be other reasons why runners with better lung function produced faster marathons; e.g., taller individuals (and those with larger lung capacities) are also likely to exhibit a longer stride length. Nevertheless, in an earlier study, Warren et al. [37] assessed lung function every 3 h in competitors of a 24-h footrace. There were significant reductions in MVV_12_ after 24 h, which were significantly related to the variance in running speed. The authors concluded that the decrease in ventilatory muscle endurance might constrain running speed during extremely long ultra-marathons. These studies provide interesting insights into lung function and its potential predictive power on endurance running performance.

Finally, although a decrease in pulmonary function appears to be a common response to marathon and ultra-marathon running, a pertinent question is whether such decreases reach levels of clinical importance (i.e., do post-race values fall below the lower-limits of normal?). Given that the majority of studies do not address this question directly, those that reported significant post-race reductions in spirometric indices were collated and subjected to further analyses. Of the initial eight studies, one did not report baseline function [66], and another reported mean data from subjects of various sexes, ethnicities, contesting both marathon and half-marathon [39], and the appropriate reference equation could not be discerned. For the six remaining studies, mean pre- to post-race data were analysed using the regression equations supplied by the Global Lung Function Initiative [67], and then compared to the most current population-specific reference values and their lower-limits of normal [68]. While care was taken to include the appropriate participant demographics and to separate data obtained from male and female runners, this was not possible in only one study (1/3 female sample) due to the non-reporting of individual subject data [40]; in this instance, the reference values for males were used so that the data could be interpreted conservatively.

The findings from these analyses are shown in Table 2, and several conclusions were drawn: (1) the largest mean pre- to post-race decrease in pulmonary function (FVC + FEV_1_) was exhibited following an ultra-marathon [29]. The subjects in that study exhibited baseline function that was slightly lower than that generally reported elsewhere (FVC = 5.0 L [94% predicted]; FEV_1_ = 3.79 L [87% predicted]); (2) immediately following the race, there was a significant decrease in both FVC (4.38 L [87% predicted]) and FEV_1_ (3.43 L [79% predicted]), both of which remained within an acceptable range of the lower-limit of normal (FVC = 4.27 L; FEV1 = 3.45 L). Relative to the other studies, these were the lowest post-race values when expressed as a percentage of predicted values (with the exception of Gordon et al., who only provided data on the FVC); (3) in all studies reassessed, the post-race FEV_1_/FVC ratio remained within acceptable limits (range 0.78–0.85), and in only one of these studies was there a noteworthy increase in the ratio [28], suggesting a degree of restriction (i.e., greater expiratory flow rates relative to lung capacity); (4) the most robust post-race lung function was observed in those groups who exhibited the strongest baseline function. Given these observations, it is reasonable to suppose that the respiratory systems of trained runners are sufficiently robust to endure most race types, providing that athletes begin the race with a healthy resting function. Although speculative, the same magnitude of response in an individual with below-average baseline parameters, or in an individual with a pre-existing respiratory disorder (e.g., asthma), may result in manifestations of clinical significance. If such a scenario presents itself, some authors have suggested that runners requiring supplemental oxygen on the basis of respiratory distress should be disqualified from competition [21]. Accordingly, pre-event respiratory screening is recommended, particularly for individuals participating in events staged in remote and/or extreme environments where medical assistance may not be readily available.

**Table 2:** Basic spirometry, for those studies reporting significant post-race reductions, reported alongside the predicted values and the lower-limits of normal

Author (s)	Date	Event	FVC L (%Pred)			FEV_1_ L (%Pred)			FEV_1_/FVC (Pred)		
			PRE	POST	LL	PRE	POST	LL	PRE	POST	LL
Gordon et al. [25]	1924	Marathon (42.2 km)	4.3 (91)	3.5 (74)*	3.8	–	–	–	–	–	–
Maron et al. [28]	1979	Marathon (42.2 km)	5.6 (106)	5.1 (97)	4.2	4.4 (102)	4.3 (99)	3.4	0.78 (0.82)	0.84 (0.82)	0.71
Mahler and Loke [29]	1981	Ultramarathon (100 km)	5.0 (94)	4.4 (82)	4.3	3.8 (87)	3.4 (79)*	3.5	0.76 (0.82)	0.78 (0.82)	0.71
Ross et al. [32]	2008	Marathon (42.2 km)	5.7 (104)	5.5 (99)	4.4	4.6 (103)	4.6 (102)	3.6	0.81 (0.82)	0.84 (0.82)	0.72
Wüthrich et al. [40]	2014	Ultramarathon (110 km)	4.2 (88)	4.1 (86)	3.8	3.8 (98)	3.5 (91)	3.0	0.90 (0.81)	0.85 (0.81)	0.70
Vernillo et al. [33]	2015	Ultramarathon (330 km)	5.2 (104)	4.7 (94)	3.9	4.1 (104)	3.7 (94)	3.1	0.80 (0.80)	0.80 (0.80)	0.69

*FVC* forced vital capacity, *FEV*_*1*_ forced expiratory volume in 1 s, *%Pred* percentage of the predicted normal value, *LL* lower-limit of normal

*Value below the lower-limit of normal. NB, data from Wüthrich et al. comprised a 1/3 female cohort, with predicted values and lower-limits assumed for males

### Limitations and Future Perspectives

There are limited data on acute pulmonary and respiratory muscle function following marathon and ultra-marathon, for several reasons. First, despite the growing popularity of ultra-marathon, participant numbers are still low relative to other sports. Moreover, runners are often reluctant to compromise their race preparation and/or recovery to volunteer for data-collection protocols, particularly when considering the long and arduous training necessary to compete. Second, endurance races, particularly ultra-marathons, are often contested in remote locations and environmental extremes, which do not lend themselves to complex or invasive data-collection protocols requiring equipment that can be rarely transported. As a result, many of the studies included in the present review are restricted to basic spirometry and handheld mouth-pressure devices, which are convenient and easily-transportable but do not provide particularly mechanistic insights. Moreover, given the transient nature of the post-race decreases in pulmonary and respiratory muscle function, complex procedures that take additional time to perform would rarely yield valid data. To gain a more comprehensive understanding of the pulmonary and respiratory muscle responses to marathon and ultra-marathon, there must be a drive to obtain more robust, high-quality data. This can be achieved by: (1) not spurning laboratory-based studies for fear of compromising ecological validity; (2) contriving to transport laboratory-based equipment into the field; and (3) using innovative study methodologies when neither of these former two can be achieved.

There are several pertinent areas of research that warrant further study. Decreases in pulmonary function following marathon and/or ultra-marathon have been reported with, and without, the presence of airway obstruction; furthermore, the magnitude of the response to any given race is highly variable. Accordingly, more research is needed to establish the characteristics that make runners susceptible to post-race decrements in pulmonary function. Where changes in function are observed, more data are needed to elucidate the causative mechanisms. There is also a growing body of research to suggest that marathon running may be associated with a mild and transient pulmonary oedema in up to half of all participants [10]; yet, to the author’s knowledge, there is but a single case-report of pulmonary oedema occurring in two runners following a 90-km ultra-marathon [69]. Further research is needed in this regard to elucidate the tolerability to races of extreme distance and duration. With respect to respiratory muscle function, a majority of studies have inferred respiratory muscle fatigue using subjective (voluntary) mouth-pressure manoeuvres, with only one study revealing the mechanisms of action using objective nerve-stimulation techniques [40]. More data are needed, specifically, to assess inspiratory and expiratory muscle fatigue following marathon/ultra-marathon running using magnetic stimulation of the phrenic and thoracic nerves. Finally, further studies are needed to clarify whether targeted respiratory muscle training has the potential to offset respiratory muscle fatigue in response to endurance running, and the extent to which this might impact on performance.

## Conclusion

Marathon and ultra-marathon appear sufficient to provoke reductions in pulmonary function in the range of 10–15%, with or without evidence of airway obstruction, and/or evidence of respiratory muscle fatigue in the range of 15–25%. Although there are no data to suggest that the post-race decreases in pulmonary function are clinically significant, there might be more severe consequences for individuals with pre-existing respiratory disorders, and the available data highlight the importance of robust baseline parameters prior to participating in a marathon or ultra-marathon. Pre-event respiratory screening is recommended, particularly for individuals participating in events staged in remote and/or extreme environments where medical assistance may not be readily available. The potential consequences of exercise-induced respiratory muscle fatigue on endurance-running performance have been discussed. The conclusions drawn from this review might influence marathon and ultra-marathon training and preparation strategies, as well as inform medical best-practice of personnel supporting the events (i.e., medics, race directors, and volunteers).
