# Correction to: Pulmonary and Respiratory Muscle Function in Response to Marathon and Ultra-Marathon Running: A Review

**DOI:** 10.1007/s40279-019-01140-7

**Published:** 2019-06-17

**Authors:** Nicholas B. Tiller

**Affiliations:** Academy of Sport and Physical Activity, Sheffield Hallam University, Collegiate Crescent, Sheffield, S10 2BP UK

## Correction to: Sports Medicine (2019) 49:1031–1041 10.1007/s40279-019-01105-w

On page 8, in the left-hand column, third paragraph, lines 9–13 which previously read:

Of the initial eight studies, one did not report baseline function [66], and another reported mean data from subjects of various sexes, ethnicities, contesting both marathon and half-marathon [39], and the appropriate reference equation could not be discerned.

Should read as:

Of the initial eight studies, one did not report absolute post-race values [66], and another reported mean data from subjects of various sexes, ethnicities, contesting both marathon and half-marathon [39], and the appropriate reference equation could not be discerned.

